# RELEASE (REdressing Long-tErm Antidepressant uSE): protocol for a 3-arm pragmatic cluster randomised controlled trial effectiveness-implementation hybrid type-1 in general practice

**DOI:** 10.1186/s13063-023-07646-w

**Published:** 2023-09-28

**Authors:** Katharine A. Wallis, Maria Donald, Mark Horowitz, Joanna Moncrieff, Robert S. Ware, Joshua Byrnes, Karen Thrift, MaryAnne Cleetus, Idin Panahi, Nicholas Zwar, Mark Morgan, Chris Freeman, Ian Scott

**Affiliations:** 1 General Practice Clinical Unit, Medical School, The University of Queensland, Herston, Brisbane, QLD 4072 Australia; 2 NHS Foundation Trust, Research and Development Department, London, Northeast London UK; 3 University College London, London, UK; 4 Griffith University, Nathan, Brisbane, QLD Australia; 5 Faculty of Health Sciences and Medicine, Bond University, Robina, Gold Coast, QLD Australia; 6 The University of Queensland, Herston, Brisbane, QLD 4072 Australia

**Keywords:** Antidepressants, Discontinuation, Deprescribing, Primary care, Withdrawal, Tapering, Long-term antidepressant use, Randomised controlled trial

## Abstract

**Background:**

Many people experience withdrawal symptoms when they attempt to stop antidepressants. Withdrawal symptoms are readily misconstrued for relapse or ongoing need for medication, contributing to long-term use (> 12 months). Long-term antidepressant use is increasing internationally yet is not recommended for most people. Long-term use is associated with adverse effects including weight gain, sexual dysfunction, lethargy, emotional numbing and increased risk of falls and fractures. This study aims to determine the effectiveness of two multi-strategy interventions (RELEASE and RELEASE+) in supporting the safe cessation of long-term antidepressants, estimate cost-effectiveness, and evaluate implementation strategies.

**Methods:**

**Design:**

3-arm pragmatic cluster randomised controlled trial effectiveness-implementation hybrid type-1. Setting: primary care general practices in southeast Queensland, Australia. Population: adults 18 years or older taking antidepressants for longer than 1 year. Practices will be randomised on a 1.5:1:1 ratio of Usual care:RELEASE:RELEASE+. Intervention: RELEASE for patients includes evidence-based information and resources and an invitation to medication review; RELEASE for GPs includes education, training and printable resources via practice management software. RELEASE+ includes additional internet support for patients and prescribing support including audit and feedback for GPs. Outcome measures: the primary outcome is antidepressant use at 12 months self-reported by patients. Cessation is defined as 0 mg antidepressant maintained for at least 2 weeks. Secondary outcomes: at 6 and 12 months are health-related quality of life, antidepressant side effects, well-being, withdrawal symptoms, emotional numbing, beliefs about antidepressants, depressive symptoms, and anxiety symptoms; and at 12 months 75% reduction in antidepressant dose; aggregated practice level antidepressant prescribing, and health service utilisation for costs. Sample size: 653 patients from 28 practices. A concurrent evaluation of implementation will be through mixed methods including interviews with up to 40 patients and primary care general practitioners, brief e-surveys, and study administrative data to assess implementation outcomes (adoption and fidelity).

**Discussion:**

The RELEASE study will develop new knowledge applicable internationally on the effectiveness, cost-effectiveness, and implementation of two multi-strategy interventions in supporting the safe cessation of long-term antidepressants to improve primary health care and outcomes for patients.

**Trial registration:**

ANZCTR, ACTRN12622001379707p. Registered on 27 October 2022.

## Administrative information

Note: the numbers in curly brackets in this protocol refer to SPIRIT checklist item numbers. The order of the items has been modified to group similar items (see http://www.equator-network.org/reporting-guidelines/spirit-2013-statement-defining-standard-protocol-items-for-clinical-trials/).

**Table:** 

Title {1}	RELEASE (REdressing Long-tErm Antidepressant uSE): protocol for a 3-arm pragmatic cluster randomised controlled trial effectiveness-implementation hybrid type-1 in general practice
Trial registration {2a and 2b}.	Title: RELEASE (REdressing Long-tErm Antidepressant uSE): Protocol for a 3-arm pragmatic cluster randomised controlled trial effectiveness-implementation hybrid type-1 in general practice. Trial ID: ACTRN12622001379707p. Australian and New Zealand Clinical Trials Registry. Date Registered: 27th October 2022. Link: https://www.anzctr.org.au/ACTRN12622001379707p.aspx
Protocol version {3}	Version 8.0 4th July 2023.
Funding {4}	Australian Commonwealth Department of Health, Medical Research Future Fund (MRFF) 2020 Clinician Researchers: Applied Research in Health - MRFAR000079. National Health and Medical Research Council (NHMRC) 2021 Partnership Projects PRC3 - 2015744
Author details {5a}	Katharine A Wallis, Mayne Professor and Head of the Mayne Academy of General Practice and Head, General Practice Clinical Unit, k.wallis@uq.edu.au; Maria Donald, Principal Research Fellow, m.donald@uq.edu.au; Karen Thrift, Practice Liaison Officer, k.thrift@uq.edu.au; Maryanne Cleetus, Project Manager, m.cleetus@uq.edu.au; Idin Panahi, Practice Liaison Officer, i.panahi@uq.edu.au **General Practice Clinical Unit, Medical School, The University of Queensland, Herston 4072, Queensland, Australia** Mark Horowitz, Clinical Research Fellow in Psychiatry, Mark.Horowitz@nelft.nhs.uk **NHS Foundation Trust, Research and Development Department, Northeast London, London, UK** Joanna Moncrieff, Professor of Critical and Social Psychiatry, j.moncrieff@ucl.ac.uk; **University College London, UK** Robert S Ware, Professor of Biostatistics, r.ware@griffith.edu.au; Joshua Byrnes, Professor of Health Economics, j.byrnes@griffith.edu.au; **Griffith University, Queensland, Australia** Nicholas Zwar, Professor of Primary Care and Executive Dean, nzwar@bond.edu.au; Mark Morgan, Professor of General Practice, mmorgan@bond.edu.au; **Faculty of Health Sciences and Medicine, Bond University, Queensland, Australia.** Christopher Freeman, Conjoint Associate Professor of Safe and Effective Medication Research, c.freeman4@uq.edu.au; Ian Scott, Professor of Medicine, Ian.Scott@health.qld.gov.au **The University of Queensland, Herston 4072, Queensland, Australia**
Name and contact information for the trial sponsor {5b}	Research Office, The University of Queensland, Cumbrae Stewart Building, The University of Queensland, Brisbane 4072.
Role of sponsor {5c}	The trial sponsor (The University of Queensland) was not involved in the study design or writing of the study protocol.

## Introduction

### Background and rationale {6a}

Antidepressant use is increasing internationally, mostly driven by increasing long-term use (> 12 months) [1–3]. Approximately 1 in 7 adults is now taking antidepressants [4], and half of users are long-term users [3]. Yet there is no reliable evidence for the benefits of long-term antidepressant use due to the short duration of acute treatment trials and methodological limitations of relapse prevention trials including confounding by withdrawal symptoms and selection of responders [5]. Clinical guidelines recommend only 6–12 months of antidepressant therapy for a single episode of moderate to severe depression and psychological therapies for anxiety and less severe depression [6–8]. There is evidence for harm with long-term use including weight gain, sexual dysfunction which may be persistent [9, 10], lethargy, emotional numbing (“reduced sympathy and empathy” [11] and “caring less about others” [12]), and increased risk of falls and fractures [13].

Many people experience unpleasant withdrawal symptoms when they attempt to stop taking antidepressants. Withdrawal symptoms include anxiety, low mood, dizziness, ‘brain fog’, irritability, headache, and insomnia, which can last for weeks or months with severity likely proportional to duration of use [14–17]. Withdrawal symptoms are often misconstrued, by both patients and doctors, as relapse or ongoing need for medication contributing to long-term use [18, 19].

Australia’s 2020 Productivity Commission Mental Health Inquiry report highlighted the problem of increasing antidepressant use and associated adverse effects and recommended as a “priority reform” to “address adverse outcomes from prescribing practices of mental health medication” (p.3)c [20]. The report stated that: “while antipsychotic prescribing in aged care facilities is one element of this … arguably a greater concern, given its frequency, is antidepressant prescribing” (p.713) [20]. Data suggest that 30–50% of long-term antidepressant users have no clinical indication for continued use and could try stopping [21].

Important factors for stopping antidepressants include reviewing the need for ongoing therapy, recognising withdrawal symptoms, slow tapering of drug dose, availability of drug mini-doses, and monitoring and reassurance to address the fear of relapse [22–24]. Non-randomised evidence supports hyperbolic tapering of drug doses to minimise withdrawal symptoms [25–28]. Hyperbolic tapering uses increasingly smaller decreases in drug dose which generates a steady decrease in brain receptor occupancy [29]. Hyperbolic tapering is now recommended in clinical guidelines [30–32].

Most antidepressant prescribing occurs in primary care general practice [4], where implementation of tapering is negligible and there is low awareness and recognition of withdrawal symptoms [6–8]. Patients report being advised to wean off antidepressants over about 4 weeks by halving their dose and then halving again [23, 33]. Further, tapering protocols and drug mini-doses are often not readily available [8, 34]. While there is the opportunity to review the need for ongoing antidepressant therapy at every repeat prescription in general practice, medication review alone is not effective for supporting the cessation of long-term antidepressants [35]. The RELEASE and RELEASE+ interventions were designed to address these factors.

The aim of this study is to determine the effectiveness of two multi-strategy interventions, RELEASE and RELEASE+, in supporting safe cessation of long-term antidepressants compared to Usual care.

### Objectives {7}

1. To determine the effectiveness of:
  - The RELEASE intervention compared to Usual care in supporting the safe cessation of long-term antidepressants
  - The RELEASE+ intervention compared to Usual care in supporting the safe cessation of long-term antidepressants
2. To estimate the cost-effectiveness of RELEASE, RELEASE+ and Usual care.
3. To evaluate the implementation of the two multi-strategy interventions.
4. To explore the effectiveness of RELEASE compared to RELEASE+ in supporting safe cessation of long-term antidepressants.

### Trial design {8}

This study was begun as a 2-arm pragmatic cluster randomised controlled trial effectiveness-implementation hybrid type-1 and pivoted, for reasons of efficiency, to a 3-arm trial design after receiving additional funding to test a more intensive version of the RELEASE intervention (RELEASE+). This transition occurred early in the trial, after only two practices had been recruited and randomised (one to RELEASE and one to Usual care).

A cluster randomised trial is being conducted, with the unit of randomisation of the primary care practice to avoid possible contamination between intervention and control arms. The unit of analysis is the individual patient [36].

## Methods: participants, interventions and outcomes

### Study setting {9}

The study setting is 28 primary care general practices in south-east Queensland, Australia. Practices will be recruited through the UQGP Research practice-based research network via email, telephone and outreach visits. We will seek to involve all or most GPs in each practice.

### Eligibility criteria {10}

Patients 18 years or older will be eligible if they are currently taking antidepressant medication and have been taking medication for longer than 12 months, including both patients who are motivated and unmotivated to stop. Antidepressants include selective serotonin reuptake inhibitors (SSRIs) (sertraline, escitalopram, fluoxetine, paroxetine, fluvoxamine and citalopram), serotonin and norepinephrine reuptake inhibitors (SNRIs) (venlafaxine, desvenlafaxine, duloxetine) and other antidepressants (mirtazapine, vortioxetine, mianserin, moclobemide, reboxetine, agomelatine). Patients will be excluded if they have a diagnosis of bipolar disorder, psychotic disorder, obsessive-compulsive disorder, substance use disorder or dementia; are currently under the care of a psychiatrist; have a non-psychiatric indication for an antidepressant (for example, neuropathic pain); are unable to give informed consent; live in residential aged care; or if the GP considers the patient not suitable for medication review.

### Who will take informed consent? {26a}

Eligible patients who have been taking antidepressants for longer than 12 months are identified through an automated search of the practice database and a list of patients is generated for each general practitioner (GP). The automated search system applies inclusion and exclusion criteria. General practitioners review the list and de-select any remaining ineligible patients and those not suitable for medication review, recording the reason for exclusion. The practice contacts patients remaining on the list via SMS, email or letter (depending on practice preference) to inform them about the study and to advise that the practice will phone to discuss the study and invite their participation, where practicable including the participant information sheet. Patients may opt out of this phone call by contacting the practice.

The practice phones patients to discuss the study. At the phone call, patients who express an interest in participating or who wish for further information have their name and email address entered into the secure research data collection tool REDCap (Research Electronic Data Capture, Vanderbilt, USA). REDCap generates an automated email to these patients with a link to the participant information sheet, consent and baseline e-survey. Patients who wish to participate may click the link and provide signed electronic consent, and then the link to complete the baseline e-survey.

### Additional consent provisions for collection and use of participant data and biological specimens {26b}

The trial does not involve collecting biological specimens for storage.

## Interventions

### Explanation for the choice of comparators {6b}

The RELEASE and RELEASE+ interventions are informed by the 3As (Ask, Advise, Assist) model of brief interventions for patient-centred care and designed to prompt and support safe cessation of long-term antidepressants using hyperbolic tapering of drug dose. Qualitative interviews with GPs [37–39], and a think-aloud study with patients with lived experience of long-term antidepressant use (under review for publication) informed intervention development. The 3As is an evidence-based behaviour change model with demonstrated feasibility in the general practice setting [40, 41]. The 3As in this context include ‘Asking’ patients how long they have been on antidepressants, and whether they are aware that clinical guidelines usually recommend only 6 to 12 months of antidepressant therapy; ‘Advising’ patients that long-term use is not harmless, that withdrawal symptoms are common but often misconstrued for relapse [18, 22, 24, 25]; and that hyperbolic tapering of drug dose can minimise withdrawal symptoms [25, 27]; and ‘Assisting’ patients by providing drug-specific hyperbolic tapering protocols and prescriptions for requisite drug mini-doses.

Control patients receive care as usual without mailout or invitation to schedule a medication review appointment. As usual, control group patients may see their GP to obtain a repeat prescription for antidepressants and may decrease or stop antidepressants at any time. This is permitted as in both arms of the trial all prescribing decisions are made as usual by GP and patient together. General practitioners in the control group receive their list of patients on long-term antidepressants for review as outlined above and receive quarterly newsletters with trial updates.

### Intervention description {11a}

RELEASE and RELEASE+ are user-informed multi-strategy interventions designed to prompt and support safe cessation of long-term antidepressants. The interventions include for patients, information about antidepressants, resources and an invitation to schedule and attend an appointment with their GP to discuss and review their use of antidepressants; and for GPs, printable resources, education and training.

For patients, RELEASE includes a study pack that is sent to patients via both email and post containing resources including the (i) medicines information brochure titled “Stopping antidepressants”; (ii) decision aid; (iii) antidepressant tapering protocol; and (iv) a letter inviting patients to schedule and attend an appointment with their GP to discuss and review their antidepressant use. Where feasible, practices reiterate this invitation by sending an SMS to patients inviting them to schedule and attend this medication review appointment. Automated email prompts are sent every 4 weeks for 6 months to remind participants to schedule and attend this review appointment. Usual costs apply to the medication review to enable scalability. Any follow-up is decided by GP and patient together.

The medicines information brochure is designed to raise awareness of clinical guideline recommendations and long-term antidepressant adverse effects, increase recognition of withdrawal symptoms, and to prompt people to reconsider their long-term antidepressant use. The decision aid is designed to help people to consider the pros and cons of continuing or stopping antidepressants and to support shared decision-making. The tapering protocol provides step-by-step guidance for hyperbolic tapering of antidepressant drug dose and advice on withdrawal symptoms including advice to return to the previous drug dose if symptoms are severe and when ready try again using an even more gradual taper. Consistent with patient-centred care, the tapering protocols are designed to be flexible with the patient in charge of tapering speed, enabling patients to taper more slowly or more quickly depending on symptoms. The invitation letter is designed to prompt medication review and a RELEASE conversation between patient and GP.

For GPs, RELEASE includes (i) an interactive e-learning module including vignettes with experts in the field; (ii) an outreach visit with in-person training in the RELEASE process including prescribing for hyperbolic tapering of antidepressants; (iii) tailoring of the practice management software to include printable drug-specific tapering protocols and customised prescriptions for requisite antidepressant drug mini-doses needed for tapering; (iv) optional comment on participant e-health record to flag study participants to GPs; and (v) a study pack containing a one-page overview of the RELEASE process and printed copies of RELEASE resources (medicines information brochure, decision aid, tapering protocol). The GPs in intervention practices may access and print out a tapering protocol and/or custom prescription through the e-health record of any patient. To reduce the risk of cross-practice contamination where GPs work in more than one practice, these resources are not available to GPs in any Usual care or non-participating practice.

Local community pharmacies near to intervention practices are also provided with a letter with information about the study and the issue of long-term antidepressant use seeking their support for patient participants who elect to taper and discontinue antidepressants.

RELEASE+ includes, in addition to the above, for patients, internet-supported access to the resources, messaging and e-mental health supports; and for GPs, additional prescribing support with continuing professional development eligible activities including clinical audit and feedback on antidepressant prescribing.

### Criteria for discontinuing or modifying allocated interventions {11b}

There are no specific criteria for discontinuing or modifying allocated interventions. The Data Monitoring and Safety Committee will monitor the progress of the trial and if they deem interim analyses or trial cessation are necessary will make this recommendation to the Investigator Committee.

### Strategies to improve adherence to interventions {11c}

Patients are sent automated reminders from REDCap to schedule and attend an appointment with their GP for medication review, as outlined above. General practitioners are sent reminders to complete the e-learning module at 2 weeks and 6 weeks.

### Relevant concomitant care permitted or prohibited during the trial {11d}

In all arms of the trial, all healthcare decisions are made as usual by GP and patient together. This includes prescribing decisions, follow-up, and referrals for example for psychological therapy.

### Provisions for post-trial care {30}

Participating patients remain under the care of their usual GP.

### Outcomes {12}

Effectiveness outcome measures are collected via e-survey at baseline, and at 6 and 12 months post patient allocation.

#### Primary outcome measure

The primary outcome is antidepressant cessation at 12 months self-reported by patients. Cessation is defined as 0 mg antidepressant maintained for at least 2 weeks.

#### Secondary outcome measures

Secondary outcomes include 75% antidepressant dose reduction at 12 months. A 75% reduction has health benefits and is likely more achievable for people who have been on antidepressants for many years [26].

##### Health-related quality of life (SF-12)

The SF-12 assesses quality of life functional health status [42]. This may detect the quality of life gained or lost related to relief of antidepressant adverse effects or an increase in antidepressant withdrawal symptoms.

##### Antidepressant side effects (ASEC-12)

The ASEC-12 is a checklist developed as part of the GENDEP (Genome-Based Therapeutic Drugs for Depression) research to measure antidepressant side effects [43]. The checklist demonstrates good agreement with psychiatrists’ interview ratings of side effects [44]. The checklist includes a question about sexual function.

##### Well-being (WEMWBS-14)

The WEMWBS-14 measures the subjective experiences of happiness and life satisfaction, and positive psychological functioning including positive relationships with others, autonomy and self-acceptance. Since long-term antidepressant use can cause emotional numbing, not caring about others, and a sense of dependence on medication, stopping antidepressants may lead to the reverse of these which may be captured by the WEMWBS scale, or alternatively a worsening in response to a loss of potential beneficial antidepressant effects. This scale has been shown to be responsive to change following mental health interventions [45].

##### Discriminatory antidepressant withdrawal symptoms (DAWS)

The DAWS scale includes 22 questions measuring symptoms that were found to be most different between before starting antidepressants and after stopping and asking patients to rate the severity of these symptoms [46]. The scale includes both physical (e.g., dizziness, electric ‘zaps’) and emotional (e.g. anxiety, worsened mood) symptoms and is used to help distinguish withdrawal symptoms from relapse, a distinction that has been problematic for antidepressant discontinuation trials [18, 47].

##### Emotional numbing (ERNS-General subscale)

The Emotional Reactivity and Numbing Scale (ERNS) was developed to measure numbing to positive stimuli and reactivity to anger or sadness for people with post-traumatic stress disorder. The general subscale of the ERNS includes eight items (e.g., I am able to feel a wide range of emotions; I feel like I am emotionally numb) scored on a 5-point Likert scale ranging from 1 ‘not at all typical of me’ to 5 ‘entirely typical of me’ [48].

##### Beliefs about antidepressants (BMQ-specific)

The Beliefs about Medications Questionnaire (BMQ) assesses commonly held beliefs about medications. The BMQ-Specific subscale is flexible in that it allows reference to specific mediations (antidepressant medication in the current context), and includes 10 items (e.g. having to take antidepressant medication worries me; my antidepressant medication protects me from becoming worse), scored on a 5-point Likert scale from 1 ‘strongly agree’ to 5 ‘strongly disagree’ [49].

##### Depressive symptoms (PHQ-9)

The PHQ-9 measures nine depressive symptoms based on the Diagnostic and Statistical Manual fourth edition criteria [50]. The PHQ-9 has high sensitivity and specificity in primary care [51].

##### Anxiety symptoms (GAD-7)

The GAD-7 is a seven-question instrument for measuring anxiety that has been validated for primary care [52, 53].

##### Antidepressant prescribing

Aggregated practice level antidepressant prescribing at 12 months compared to baseline are extracted from practice management systems using a data extraction tool.

Antidepressant prescriptions at 12 months from participant practice e-health record.

*Costing measures:* The primary outcome will be the incremental net monetary benefit, defined as the incremental benefit, measured in quality-adjusted life years (QALYs) multiplied by the threshold value of a QALY minus the incremental cost from a health sector perspective. Secondary analysis will also be undertaken from a broader societal perspective. The cost of the intervention will be estimated based on resource use and implementation data collected during the trial (including educator and doctor time, costs for RELEASE and RELEASE+ resources and website, practice nurse time for search, GP time to review list, and cost of compounding capsules). A cost questionnaire will be administered to participants to collect data on health and social service resource use, out-of-pocket spending, and time off work. A review of GP records will be conducted to extract additional health service usage, including medication, GP consultations, hospitalisations, outpatient appointments, and emergency department attendances.

*Implementation outcome measures:* Consistent with a type 1 effectiveness-implementation trial, we will conduct a concurrent theory-informed multi-method evaluation to assess implementation determinants (barriers and enablers) and implementation outcomes. This includes interviews, brief e-surveys and study administrative data. Semi-structured interviews with up to 40 GPs and patients will be guided by the five domains of the Consolidated Framework for Implementation Research to identify determinants crucial for understanding the context for successful implementation including what happens and why [54]. This will optimise our likelihood of effecting change through the identification and resolution of actionable barriers and enhancement of enablers (e.g. whether GPs consider RELEASE and RELEASE+ a ‘good fit’ for practice workflows in routine practice; and GPs and patient participants’ perspectives on the content and appropriateness of the resources). A bespoke database will be used to record implementation outcomes including adoption (e.g., number of practices and GPs approached to take part, and taking part; number of patients de-selected by GPs and why; and proportion of eligible patients that enrol); and fidelity to intervention (e.g., proportion of GPs who engage with the interactive e-learning module; and number of patients who follow the drug-specific tapers as evidenced by clinical notes and self-report).

### Participant timeline {13}

**Fig. 1:**
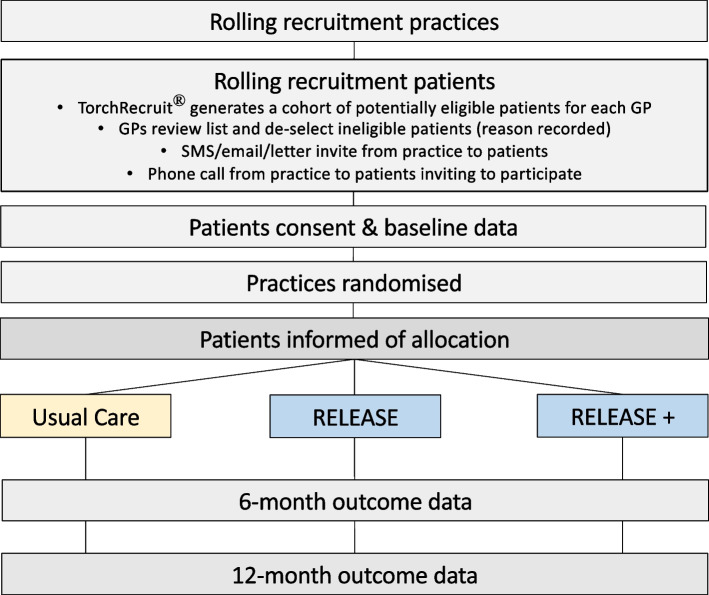
Practice and participant flow through the RELEASE study

**Fig. 2:**
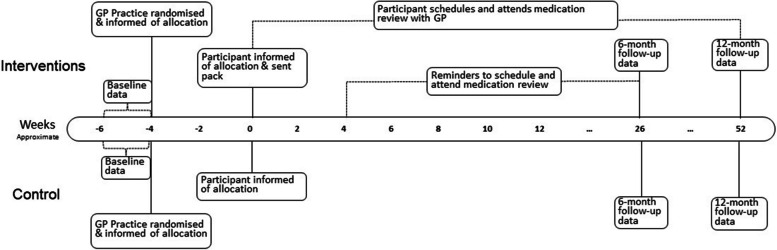
Practice and participant timeline for the RELEASE study

The study flow is set out in Fig. 1 and the participant timeline in Table 1 and Fig. 2.

**Table 1:**
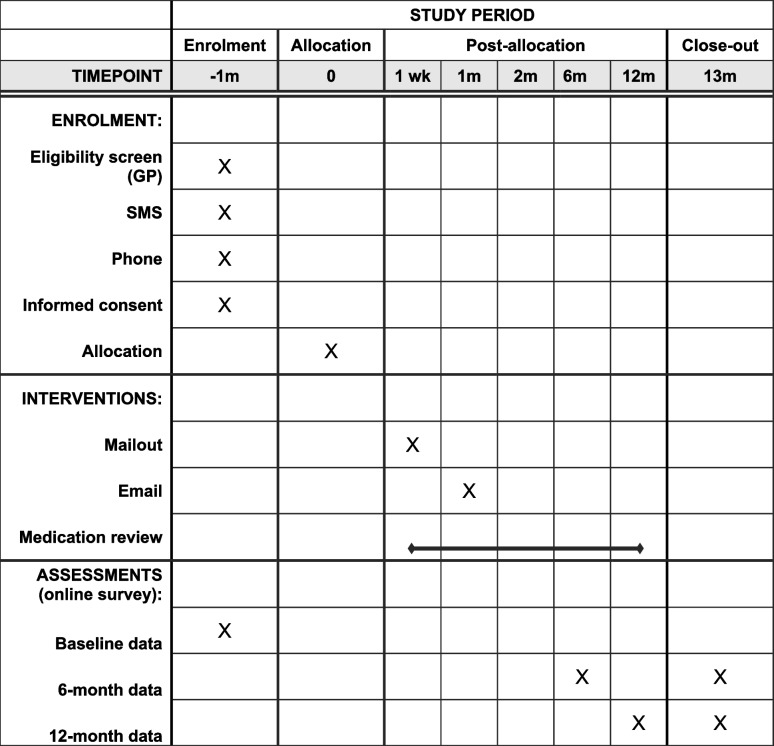
SPIRIT figure for RELEASE study schedule of enrolment, intervention, and data collection

### Sample size {14}

Based on a 2020 trial, we assume 12% of participants in the control group and 30% of participants in each of the intervention groups will achieve the primary outcome (stopped antidepressants by 12 months) [55]. Because the study involves two comparisons, Usual care vs RELEASE and Usual care vs RELEASE+, we will allocate more participants to the Usual care arm as this increases the overall efficiency of the trial (that is, for fixed type I and type II errors we require fewer total participants). The ratio chosen for allocation is 1.5:1:1. Using a two-sided alpha significance level of 0.035 (chosen to facilitate comparison of the two intervention arms against a single control arm) and 90% power then, without accounting for clustering, we would require 142 participants in the Usual care arm and 95 in each of the intervention arms (332 total).

As this is a cluster RCT, we need to account for the non-independence of observations from individual participants recruited from the same practice. We assume there will be a mean of 20 participants recruited from each practice who will provide data on the primary outcome, and that the within-practice intra-cluster correlation = 0.03, giving a design effect of 1.57. Consequently, we require 522 participants to provide data. To achieve this number, and to account for the possible drop-out of practices once enrolled, we will recruit 28 practices. Assuming 20% attrition, we require 653 participants to enrol and provide baseline data. This requires the 28 practices to recruit an average of 23.3 patients.

Conservatively estimating each practice to have on average 4000 adult patients, of whom 1 in 10 are on antidepressants (SSRIs, SNRIs, and other antidepressants) (400 patients per practice), half of them long-term (200 patients per practice), of whom an estimated 60% (120) will be identified on automated search as potentially eligible to participate in the RELEASE study. Of these potentially eligible patients, we expect GPs to deselect 30% as not suitable for medication review leaving 84 eligible patients per practice for invitation. To achieve recruitment of 653 participants requires each of the 28 practices to enrol 28% of the 84 invited eligible patients (2352 invited in total).

### Recruitment {15}

Participants are recruited via the practice. As outlined above, eligible patients on long-term antidepressants are identified through an automated search of the practice database and a list of potentially eligible patients is generated for each GP. The automated search system is compatible with the two most commonly used practice management software systems in Australia (BestPractice, Medical Director). General practitioners review the list and de-select ineligible patients and those not suitable for medication review, recording the reason for exclusion. The practice contacts eligible patients via SMS, email or letter (depending on practice preference) including the participant information sheet where possible, to inform patients about the study and to advise that the practice will phone to discuss the study and invite their participation. Patients may opt out of this phone call by contacting the practice. The practice phones patients to discuss the study.

At the phone call, patients who express an interest in participating or who wish to receive further information have their name and email address entered into the secure research data collection tool REDCap (Research Electronic Data Capture, Vanderbilt, USA). REDCap generates an automated email to these patients with a ‘closed’ link (i.e., specific to the patient’s email) with the Participant Information Sheet, and a link to consent and the baseline e-survey. Patients who decide to participate click the link to provide signed electronic consent. They may then complete the baseline e-survey.

The REDCap system generates an automated email on days 3, 6 and 9 to participants who have provided consent to remind them to complete the baseline e-survey. At day 14 (from the phone call), the baseline survey is closed to participation. The contact details of patients who have not completed the survey, not provided consent, or decided not to participate are erased from REDCap.

## Assignment of interventions: allocation

### Sequence generation {16a}

Randomisation is clustered at the practice level to avoid contamination between intervention and Usual care arms. Practice randomisation occurs after baseline data collection is closed for each practice to avoid bias in GP de-selection. Randomisation occurs in a 1.5:1:1 Usual care:RELEASE:RELEASE+ ratio via a central web-based randomisation service. Practices are stratified by size (> 5/ ≤ 5 FTE GPs). Randomisation occurs in blocks randomly assigned in a 1:1 ratio.

The first two recruited practices were randomised into one of two arms (one was allocated to Usual care and one to RELEASE). Thereafter, the trial transitioned from a 2-arm to a 3-arm cluster RCT as outlined above.

### Concealment mechanism {16b}

The allocation sequence is generated by a central web-based randomisation service separate to the research recruitment team.

### Implementation {16c}

The research recruiting team recruit the practices and email to the randomisation service the practice identifier and size (FTE GPs). The randomisation service notifies the research team of practice allocation via email. The research team notify the practice of allocation. After a 4-week run-period that allows time to prepare GPs and practices in intervention arms, patients in all groups are informed of allocation.

## Assignment of interventions: blinding

### Who will be blinded {17a}

Both GPs and patients are blinded until after baseline data has been collected. Randomisation of practices occurs after baseline data is closed for each practice. Given the cluster trial design and the nature of the interventions, it is not possible for patients and GPs to be blinded after allocation. Self-reported outcome measures are used to prevent researcher/observer rating bias. The statisticians and health economists analysing the data will be kept blind to allocation. Unblinded researchers will obtain information from participants’ practice e-health records after the study ends and 12-month e-survey data collection is complete.

### Procedure for unblinding if needed {17b}

The statisticians and health economists analysing the data will be unblinded to allocation after the primary analysis is complete.

## Data collection and management

### Plans for assessment and collection of outcomes {18a}

Data collection at baseline, 6 and 12 months are through online e-survey following email invitation from the REDCap data management software. At the end of the study, a researcher extracts health service utilisation data from participants’ practice e-health records including the number of consultations and use of health services outside the practice such as hospitalisations and emergency department visits.

### Plans to promote participant retention and complete follow-up {18b}

Patient participants receive AU$40 e-voucher for completing each e-survey (at baseline, 6 and 12 months). Participants are sent an email reminder on days 3, 6, and 9 to complete the baseline e-survey, and 3 × weekly reminders to complete the 6- and 12-month e-surveys. If the patient has not been contactable after 4 weeks, they are deemed to have been lost to follow-up at that point. The same approach is repeated at 12 months, even if the patient has been deemed lost to follow-up at 6 months. If necessary, practices will SMS participants to prompt follow-up data collection.

### Data management {19}

Participant data are entered directly into a secure REDCap database. The University of Queensland’s REDCap server provides a web interface using the industry-standard SSL (secure sockets layer) (SHA256 RSA 2048 bit keys) to ensure data privacy. Access to the REDCap service is via the UQ network using SSL through the University's firewalls. Login usernames are allocated to researchers by the REDCap administrative staff and passwords are managed by the REDCap system and the user. De-identified datafiles are stored on the secure University of Queensland Research Data Manager (UQRDM) with access restricted to the relevant research team members. UQRDM complies with the Australian Code for the Responsible Conduct of Research.

### Confidentiality {27}

Each practice and each participant are assigned a unique study identifier. Participant details will remain confidential. Participants’ names will not be recorded outside the university. The patient consent form specifies the data to be collected and how the data is managed and used. Access to the electronic data in REDCap is limited to members of the research team with appropriate training who require access. De-identified data will be used in data analysis.

### Plans for collection, laboratory evaluation and storage of biological specimens for genetic or molecular analysis in this trial/future use {33}

The trial does not involve the collection of biological specimens for storage.

## Statistical methods

### Statistical methods for primary and secondary outcomes {20a}

The full statistical analysis plan will be finalised prior to the completion of recruitment and will be uploaded to an open-access repository. The unit of analysis is the individual patient. Analyses will account for the clustering at the practice level.

Outcomes will be analysed on an intention-to-treat basis. We may also present a per-protocol analysis. Summary statistics will be described using either mean (standard deviation) or median (25th–75th percentile) for continuous variables, according to distribution, or as frequency (percentage) for categorical variables.

The primary outcome will be assessed using mixed-effects logistic regression, with effect estimates presented as odds ratio and 95% confidence interval (95% CI). The treatment group (Usual care/RELEASE/RELEASE+) will be included as the main fixed effect and the practice will be included as a random effect to account for probable non-independence of results from participants who attend the same practice. The primary outcomes of interest are the between-group comparisons of RELEASE versus Usual care and RELEASE+ versus Usual care. Secondary outcomes will similarly be analysed using mixed-effects models, using linear regression for interval outcomes, logistic regression for binary outcomes, and Poisson regression for count outcomes. All models will include the practice as a random effect. Longitudinal associations will be investigated using analyses that account for the multiple observations per participant, with the particular analysis determined by data structure, for example, three-level hierarchical mixed-effects models with ‘patient’ nested within ‘GP’ nested within ‘practice’ where appropriate. Regarding missing data, sensitivity analyses will be conducted using imputation models. Patients who drop out before assessment will be classified as ‘not able to stop’.

Differences between groups in total cost for the within-trial analysis will be assessed using a general linear model (gamma family, log link). QALYs will be estimated for the within-trial analysis based on responses to the SF-12 Short Form QoL instrument and Australian utility algorithm using an area under the curve approach and a base case threshold value of $50,000, as this is a commonly cited threshold value in Australia. Differences between groups will be tested using a student t-test where the assumption of normal distribution holds and generalised linear models in the case of non-normally distributed data. Multi-level regression analysis to correct for correlation of error at each cluster will be used to characterise uncertainty around the estimated incremental net monetary benefit for the within-trial analysis. A Markov model will also be used to estimate the costs and QALYs over a patient’s lifetime under a range of extrapolation scenarios. The determination of whether RELEASE or RELEASE+ represents the greatest value for money will be determined based on the ranking of their incremental net monetary benefit values. A cost-effectiveness acceptability curve (CEAC) will assess the probability of RELEASE and RELEASE+ as the most cost-effective option over a range of threshold values.

### Interim analyses {21b}

No a priori interim analyses are planned. The Data Monitoring and Safety Committee will review safety data regularly during the trial. There may be an interim analysis at 6 months.

### Methods for additional analyses (e.g. subgroup analyses) {20b}

No a priori subgroup analyses are planned. There may be a sensitivity analysis excluding the first two practices that were recruited and randomised as part of a 2-arm trial, prior to the trial pivoting to a 3-arm trial design. There may also be sub-group analyses of primary outcome by drug class, duration of use (< 2 years, 2–5 years, > 5 years), and possibly of average dose (low, moderate, high).

Interviews will be analysed using a combination of inductive and deductive coding, beginning with an emphasis on barriers and facilitators and expanding to measure implementation outcomes including acceptability and appropriateness.

### Methods in analysis to handle protocol non-adherence and any statistical methods to handle missing data {20c}

The structure and pattern of missing data will be assessed and if necessary, a sensitivity analysis undertaken based on imputed data using a multiple imputation model.

### Plans to give access to the full protocol, participant-level data and statistical code {31c}

Anonymised quantitative (survey) datasets generated during this study may be available upon request from KW from 1 Nov 2027, depending on the types of analyses planned and the submission of a peer-reviewed, funded and ethically approved proposal. The trial dissemination group (KW and MD) oversees planned outputs from the trial and agrees on whether data are shared.

## Oversight and monitoring

### Composition of the coordinating centre and trial steering committee {5d}

An Investigator Committee chaired by KW includes all investigators and representatives from the study team. The role of the committee is to provide academic oversight of the trial, provide advice to the study team including on methodology and research outcomes; and provide comment on new research, including international research, relevant to the trial. The Committee meets via videoconference (Zoom) two times per year and as needed.

The Trial Steering Committee is chaired by KW and includes representatives with expertise in general practice, psychiatry, psychology, statistics, health economics and representatives from primary care stakeholder organisations and practices, and two consumer representatives with lived experience of long-term antidepressant use. The role of the Steering Committee is to assist in achieving outcomes and strategic goals, inform strategic directions, guide dissemination, and support scale-up. The Committee meets two to three times per year via videoconference (Zoom).

The study team manages the day-to-day running of the trial. The study team includes the clinician lead (KW), implementation lead (MD), project manager (MC), administrative assistant, practice liaison officers (KT, IP), and health services researchers. The team meets regularly face-to-face in the General Practice Clinical Unit at The University of Queensland to review progress and monitor the conduct and management of the study.

### Composition of the data monitoring committee, its role and reporting structure {21a}

The Data Monitoring and Safety Committee consists of the Clinician Lead (chair), Implementation lead, academic psychiatric trainee, practice liaison officer, health services researcher and other study team members as necessary. The Committee will meet at least two times per year and as necessary. The Committee will monitor and review outcome and safety data and submit recommendations to the trial Investigator Committee on the continuation of the trial.

### Adverse event reporting and harms {22}

The GPs will record any adverse event on a proforma incident reporting form, or via email or phone. Any report will include the nature of the event, the date of the event and the report, seriousness of event, corrective action, outcome, and likelihood of the event being related to the trial. Adverse events will be reported to the Data Monitoring and Safety Committee who will advise the Investigator Committee who will decide on whether interim analysis is necessary. Any reports of serious adverse events will be made to the ethics committee within 2 weeks.

Any patient participant who records a score of 15 or greater on the GAD-7 [52] and/or a score of 15 or greater on the PHQ-9 [50] and/or above 1 (i.e. 2 or 3) on the ninth question of the PHQ-9 survey (about suicide/self-harm) at any of the three data collection time points, will receive an automated message from REDCap advising that their score is in the range where it is recommended that they schedule an appointment to see their doctor.

### Frequency and plans for auditing trial conduct {23}

There is no external audit planned.

### Plans for communicating important protocol amendments to relevant parties (e.g. trial participants, ethical committees) {25}

Important amendments to the protocol will be discussed with co-investigators before decided. Ethical approval for agreed amendments will be sought from The University of Queensland Human Research Ethics Committee and then the trial registry will be updated.

### Dissemination plans {31a}

The trial dissemination group comprises KW and MD who oversee planned outputs from the trial. The trial results will be disseminated to participating practices and participants in summary form and will be published in peer-reviewed journals. The trial will be presented at conferences including primary care research conferences. Summary trial results will be available on the university trial website. Results will also be disseminated through networks.

## Discussion

This study transitioned from a 2-arm trial to a 3-arm trial subsequent to receiving additional funding. The 3-arm trial enables us to test both a more and less intensive intervention for supporting the safe cessation of long-term antidepressants when there is no clinical indication for continued use. The study will develop new evidence on the (i) effectiveness of the RELEASE and RELEASE+ multi-strategy interventions in supporting the safe cessation of long-term antidepressants, (ii) implementation in practice and potential for scale-up, and (iii) cost-effectiveness. This new knowledge is applicable to primary healthcare systems internationally to address increasing long-term antidepressant use to improve health outcomes.

## Trial status

This paper is based on protocol version 8, dated 4th July 2023. Version 1 of the protocol was approved by The University of Queensland Human Research Ethics Committee (2022/HE001667) on 18 Oct 2022 for 3 years. Recruitment of practices began on 1 Nov 2022 and is ongoing. Patient recruitment started on 8 March 2023 and is ongoing. Patient recruitment is expected to be completed by 29 Feb 2024. The end of the study is defined as the date of the 12-month survey data collection for the last patient, which is expected 13 months after the last practice is randomised.

## Data Availability

Anonymised quantitative (survey) datasets generated during this study may be available upon request from KW from 1 Nov 2027, depending on the types of analyses planned and the submission of a peer-reviewed, funded and ethically approved proposal. The trial dissemination group (KW and MD) oversees planned outputs from the trial and agrees on whether data are shared.

## Abbreviations

ASEC-12: 12-Item Antidepressant Side Effect Checklist
BMQ-specific: Beliefs about Medications Questionnaire: Specific subscale
CEAC: Cost-Effectiveness Acceptability Curve
CI: Chief Investigator
DAWS: Distinctive Antidepressant Withdrawal Scale
ERNS-8: Emotional Reactivity Numbing Scale: General subscale
FTE: Full-time equivalent
GAD-7: Seven-item Generalised Anxiety Disorder measure
GP: General practitioner (primary care physician)
PHQ-9: Nine-item Patient Health Questionnaire
QALY: Quality-adjusted life year
REDCap: Research Electronic Data Capture
RELEASE: REdressing Long-tErm Antidepressant uSE
SF-12: 12-Item Short-Form health survey
WEMWBS: Warwick–Edinburgh Mental Well-Being Scale
